# Cognitive trajectories following onset of psychosis: a meta-analysis – CORRIGENDUM

**DOI:** 10.1192/bjp.2022.165

**Published:** 2023-01

**Authors:** Andrew J. Watson, Lauren Harrison, Antonio Preti, Til Wykes, Matteo Cella

The authors regret the inclusion of an error in the above article.

In the references section, reference 24 is incorrect and should read as follows:

*Torgalsbøen AK, Mohn C, Czajkowski N, Rund BR. Relationship between neurocognition and functional recovery in first-episode schizophrenia: Results from the second year of the Oslo multi-follow-up study. Psychiatry Research. 2015;227(2–3):185–91.*

This error has no bearing on the results of the analysis, data reported, or interpretation of the findings.

